# Ship Detection in Synthetic Aperture Radar Images under Complex Geographical Environments, Based on Deep Learning and Morphological Networks

**DOI:** 10.3390/s24134290

**Published:** 2024-07-01

**Authors:** Shen Cao, Congxia Zhao, Jian Dong, Xiongjun Fu

**Affiliations:** 1 Beijing Institute of Technology, Beijing 100081, China; 3220210565@bit.edu.cn (S.C.); zcx09890989@163.com (C.Z.); 2 Tangshan Research Institute of BIT, Tangshan 063007, China

**Keywords:** Synthetic Aperture Radar (SAR), ship images, deep learning, object detection, morphological networks

## Abstract

Synthetic Aperture Radar (SAR) ship detection is applicable to various scenarios, such as maritime monitoring and navigational aids. However, the detection process is often prone to errors due to interferences from complex environmental factors like speckle noise, coastlines, and islands, which may result in false positives or missed detections. This article introduces a ship detection method for SAR images, which employs deep learning and morphological networks. Initially, adaptive preprocessing is carried out by a morphological network to enhance the edge features of ships and suppress background noise, thereby increasing detection accuracy. Subsequently, a coordinate channel attention module is integrated into the feature extraction network to improve the spatial awareness of the network toward ships, thus reducing the incidence of missed detections. Finally, a four-layer bidirectional feature pyramid network is designed, incorporating large-scale feature maps to capture detailed characteristics of ships, to enhance the detection capabilities of the network in complex geographic environments. Experiments were conducted using the publicly available SAR Ship Detection Dataset (SSDD) and High-Resolution SAR Image Dataset (HRSID). Compared with the baseline model YOLOX, the proposed method increased the recall by 3.11% and 0.22% for the SSDD and HRSID, respectively. Additionally, the mean Average Precision (mAP) improved by 0.7% and 0.36%, reaching 98.47% and 91.71% on these datasets. These results demonstrate the outstanding detection performance of our method.

## 1. Introduction

Synthetic Aperture Radar (SAR) is capable of generating high-resolution microwave imagery across all weather conditions and at all times. The application of SAR imagery for ship detection has become increasingly vital in domains such as maritime surveillance and navigation [1,2,3,4]. However, SAR images of ships are significantly affected by speckle noise, and complexities like coastlines, ports, and islands often complicate the detection process in certain scenarios. Therefore, high-precision ship detection in SAR imagery continues to encounter substantial challenges.

Traditional methods for ship detection using Synthetic Aperture Radar (SAR) primarily rely on the statistical attributes of targets [5], their scattering characteristics [6], and features derived from transform domains [7]. Detection is enhanced through the utilization of Constant False Alarm Rate (CFAR) techniques [8], which adjust local detection thresholds in accordance with the statistical properties of the background clutter, thereby maintaining a consistent false alarm rate. This approach is effective for identifying ships in offshore scenarios. However, there is a higher likelihood of false positives in more complex environments, such as in coastal and port regions, where the background characteristics significantly differ from the undisturbed maritime setting.

Deep learning-based object detection algorithms are generally categorized into single-stage and two-stage approaches. Single-stage methods, exemplified by YOLO [9], SSD [10], and RetinaNet [11], prioritize efficiency by processing images in a single pass. Conversely, two-stage methods, evolving from RCNN [12] to Faster R-CNN [13], enhance accuracy through an initial generation of potential object regions followed by their refinement. These deep learning techniques automatically extract image features via neural networks, accessing deeper structural attributes and high-dimensional semantic information. This capability significantly improves detection accuracy in various contexts. However, the majority of these methods are designed for optical imagery. Given the distinct imaging mechanisms and target characteristics found in Synthetic Aperture Radar (SAR) compared to optical imaging, SAR ship images often present more complex backgrounds. Consequently, many existing algorithms cannot fully adapt to SAR ship detection.

Addressing the issue of complex backgrounds, Hu et al. [14] introduced BANet, a network that accommodated the diversity in ship sizes and rotational angles. BANet enhances local information acquisition through the incorporation of deformable convolutions to build a Local Attention Module (LAM). Additionally, it employs a Non-Local Attention Module (NLAM) to boost the extraction of non-local features. This design effectively balances the extraction of local and non-local features, thereby enhancing ship detection accuracy in complex scenes. Li et al. [15] proposed a multidimensional domain deep learning network for SAR ship detection. By utilizing a polar coordinate Fourier transform, this network merges spatial and frequency domain features, leveraging multidimensional domain information to improve the detection accuracy of rotating ships against complex backgrounds. These innovative structures designed specifically for the SAR ship detection task significantly enhance detection precision. Additionally, Xu et al. [16] have proposed a lightweight airborne SAR ship detector, Lite-YOLOv5, based on the YOLOv5 algorithm, which achieves rapid ship detection without sacrificing accuracy.

Deep learning techniques excel in automated feature extraction, yet they demand substantial volumes of accurately annotated data and exhibit limited interpretability. In contrast, traditional image processing algorithms, while generally less performant, provide robust interpretability and superior computational efficiency. Merging traditional image processing methods with deep learning has been a hotspot in recent years. Qin et al. [17] proposed a multi-level wavelet speckle reduction network (Wavelet-SRNet) specifically designed for target recognition in noisy SAR images. This network integrates dual wavelet denoising branches with the original noisy imagery to effectively mitigate speckle noise. Jiang et al. [18] implemented non-subsampled Laplacian pyramid decomposition (NSLP) to extract ship morphological features directly from raw images, creating new image channels that blend with the original data to enhance ship contours while simultaneously diminishing speckle noise effects. Ai et al. [19] introduced a convolutional neural network that utilizes a multi-scale rotation-invariant Haar-like (MSRI-HL) feature set (MSRIHL-CNN). This feature set captures multi-scale, rotation-invariant textural and edge details not typically detected by conventional CNNs. These approaches synthesize the strengths of traditional image processing and deep learning, enhancing the overall effectiveness of the algorithms. The authors in [20] developed a morphological network that married traditional morphological operations with convolutional kernels. This network facilitates the training of morphological structuring elements through backpropagation, effectively eliminating raindrops of any shape from images. These approaches synthesize the strengths of traditional image processing and deep learning, enhancing the overall effectiveness of the algorithms.

To significantly enhance ship detection capabilities in complex SAR imagery environments, this study pioneers an advanced approach by amalgamating deep learning techniques with morphological networks. The innovative contributions of this research are extensively detailed as follows:

- Development of an Advanced Preprocessing Module: Combining morphological algorithms with neural networks enhances the algorithm’s generalization performance while retaining the benefits of morphological processing. By investigating the strengths and weaknesses of different morphological processing algorithms, advantageous methods are integrated into a morphological preprocessing module. This module effectively reduces noise, extracts edges, and enhances ship detail features, ensuring more effective and reliable analysis by subsequent deep learning models. The enhanced images, along with the original images, are input into the feature extraction network to maintain a comprehensive data perspective.
- Integration of the Coordinate Channel Attention (CCA) Module: In the core architecture of the detection network, a Coordinate Channel Attention (CCA) module has been implemented. This module merges the benefits of both coordinate and channel attention, simultaneously applying attention across the image’s horizontal and vertical coordinates as well as its channels. This enhancement significantly boosts the network’s sensitivity to the positioning and pertinent features of ships, thereby improving its recognition and localization capabilities. Such advancements are essential for sustaining detection accuracy in the dynamic and varied conditions of maritime environments, effectively minimizing the incidence of missed detections.
- Establishment of a Four-layer Bidirectional Feature Pyramid Network (FBFPN): FBFPN builds on the foundation of PAFPN by incorporating larger feature maps, creating a four-level feature pyramid structure specifically designed to capture detailed characteristics of ships across various scales. Furthermore, to minimize noise interference in these large-sized feature maps, features are prevented from propagating upwards. This pyramid structure enhances the precision of feature extraction, enabling the network to identify ships with increased accuracy and improved robustness against complex background noise and environmental disturbances.

Together, these advancements forge a robust framework that significantly elevates the performance of ship detection systems in SAR imagery, making them more adept at operating within challenging and cluttered maritime environments.

## 2. Methodology

**Figure 1:**
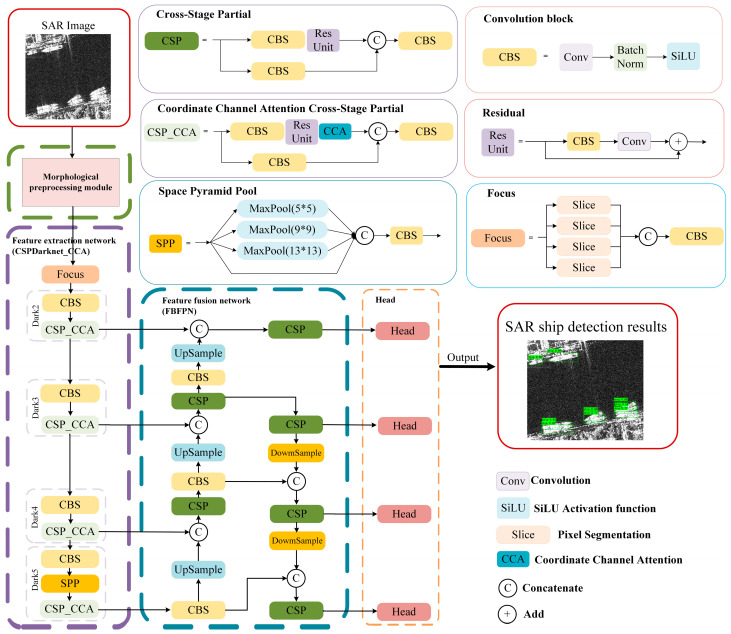
SAR ship detection model based on deep learning and morphological network.

The SAR ship detection model developed in this study is depicted in Figure 1 and consists of two main components: a morphological preprocessing module (highlighted with a green dashed line) and a comprehensive detection network. The detection network is further subdivided into three functional sections: a feature extraction network designated as CSPDarknet_CCA (illustrated with a purple dashed line), a feature fusion network called FBFPN (shown with a blue dashed line), and a detection head (outlined with an orange dashed line). Initially, the input image is subjected to a preprocessing routine involving several morphological operations, aiming at minimizing noise and enhancing critical ship features such as edges and fine details. This preprocessed image, along with the original, is subsequently introduced into the backbone network for feature extraction. This phase incorporates a Coordinate Channel Attention (CCA) module, which facilitates the generation of feature maps at varying resolutions, integrated into the CSPDarknet framework to form the CSPDarknet_CCA network as depicted in Figure 1 Following feature extraction, a sophisticated four-layer bidirectional feature pyramid network (FBFPN) is employed. This network performs feature fusion utilizing both top-down and bottom-up approaches to produce predictive feature maps. Finally, the detection head utilizes these maps to determine the positions and class confidence of the predicted bounding boxes. The ensuing sections provide an in-depth discussion of these pivotal network modules.

### 2.1. Architecture of the Morphological Network

#### 2.1.1. Fundamental Morphological Operations

Classical morphological processing involves dilation and erosion. SAR images, which characterize the backscattering properties of targets, are represented as single-channel grayscale images. Let f(h,w) denotes the original SAR image and b(x,y) the morphological structuring element. The dilation and erosion operations are defined by Equations (1) and (2), respectively, as follows:

(1) $$g_{1}=(f⊕b)(h,w)=\max\{f(h-x,w-y)+b(x,y)|(h-x),(w-y)\in D_{f},(x,y)\in D_{b}\}$$

(2) $$g_{2}=(f\Theta b)(h,w)=\min\{f(h+x,w+y)-b(x,y)|(h+x),(w+y)\in D_{f},(x,y)\in D_{b}\}$$

where ⊕ represents dilation, Θ represents erosion, and $D_{f},D_{b}$ denote the value ranges of f and b respectively.

The shape and value attributes of morphological structuring elements exert considerable influence on the outcomes of image processing applications. Conventionally, kernel functions possess predetermined shapes and values, including elliptical, rectangular, and cross-shaped forms. The selection of these kernels typically relies on empirical judgment, which often fails to adapt to the intricate scenarios encountered in SAR ship imagery. Motivated by developments in deep learning, this research innovates by converting morphological structuring elements into trainable parameters. These parameters can be initially randomized and subsequently refined through backpropagation. This transformative approach enables the morphological module to autonomously adjust aspects such as orientation and boundary thickness to meet the specific demands of ship detection tasks, thus significantly enhancing the module’s capability to conform to ship contours and intricate background conditions. The morphological module described herein is composed of layers featuring these adaptable and trainable morphological operations.

#### 2.1.2. Morphological Preprocessing Module

Morphological processing, through the strategic combination of dilation and erosion, facilitates the design of various operations tailored for tasks such as feature extraction and edge detection, thereby enabling the quantification and analysis of shapes within images. In this study, the suite of morphological operations utilized encompasses the opening, top-hat transformation, black-hat transformation, and morphological gradient. These operations are critical for enhancing the detection and characterization of structural details in images.

The opening operation is defined as the cascade of an erosion followed by a dilation applied to an image. If we denote the image by f and the structuring element by b, then the opening operation can be expressed by Equation (3):

(3) $$g_{3}=f\Theta b_{1}⊕b_{2}$$

where $b_{1}$ and $b_{2}$ denote distinct morphological structuring elements that are trained through the network. The opening operation, characterized by an erosion followed by a dilation, serves to effectively eliminate the noise of small dimensions and facilitates the segregation of closely situated ship targets. Nonetheless, this operation might induce a reduction in the apparent size of the ships, particularly affecting smaller vessels, which could adversely impact the accuracy of the detection process.

The closing operation is defined as a dilation operation followed by an erosion operation. If we denote the image by f and the structuring element by b, then the closing operation can be expressed by Equation (4):

(4) $$g_{4}=f⊕b_{1}\Theta b_{2}$$

where $b_{1}$ and $b_{2}$ denote distinct morphological structuring elements that are trained through the network. The closing operation can effectively fill small holes and cracks in the ship images, improving the integrity of the ships. However, in dense ship scenes, excessive closing operations may cause adjacent ship targets to merge, making it difficult to distinguish individual targets, thereby affecting the accuracy of detection and recognition.

The top-hat transformation, alternatively referred to as the white-hat transformation, is executed by computing the difference between the original image and the result of its opening operation. This method is particularly effective for highlighting small objects, details, or noise elements that are brighter than their immediate environment. The mathematical representation of the top-hat operation is provided in Equation (5):

(5) $$g_{5}=f-(f⊕b_{1}\Theta b_{2})$$

The top-hat transformation effectively enhances the fine features of ships and their appendages, such as masts and antennas, which significantly contributes to the accuracy of ship detection and classification. Although this operation is proficient in emphasizing small, brightly illuminated ships or details, it also has the potential to amplify noise present in the image. This is especially problematic in the cases of suboptimal image quality or elevated noise levels.

Black-hat transformation is achieved by the difference between the closing operation and the original image and is used to enhance the dark details in the image. The black-hat transformation can be described by Equation (6):

(6) $$g_{6}=(f\Theta b_{1}⊕b_{2})-f$$

The black-hat transformation enhances local contrast in images, thereby improving the visibility of dark details in ships, making the contrast between ships and the water surface more pronounced, which aids subsequent ship detection efforts. However, the black-hat transformation may also amplify image noise, especially in dark areas, which can interfere with the detection of ships.

Morphological gradient is typically achieved by calculating the difference between the dilation and erosion of an image. If f represents the image and b represents the structuring element, the morphological gradient can be described by Equation (7):

(7) $$g_{7}=(f⊕b_{1})-(f\Theta b_{2})$$

The morphological gradient effectively accentuates the edge delineations of maritime vessels, enhancing the demarcation between vessels and the surrounding background or other entities. This facilitation is advantageous for the recognition and detection of ships. Nevertheless, in imagery where the signal-to-noise ratio is suboptimal, edge detection may result in discontinuities along the boundaries, complicating the continuous identification of complete vessel perimeters and thereby undermining the efficacy of detection processes. Furthermore, as edge detection predominantly concentrates on the external contours of objects, the salient intra-ship features (e.g., structural details of decks) tend to be neglected, which could significantly contribute to the accuracy of ship detection and classification.

**Figure 2:**
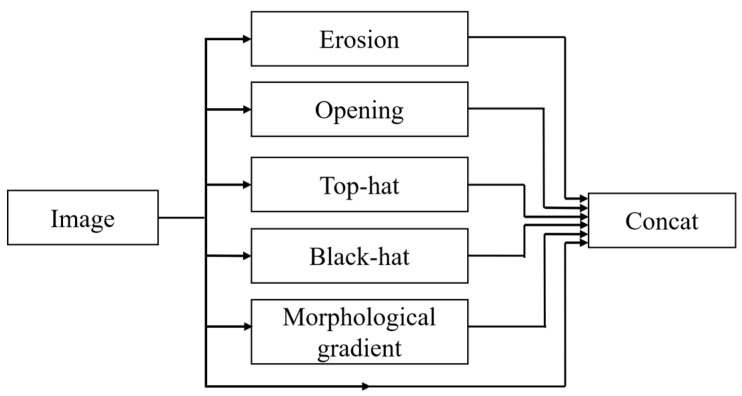
Morphological Preprocessing Module.

The diverse types of morphological operations applied to ship imagery accentuate distinct dimensions of information. This study integrates the advantages of various morphological operations by incorporating them into the network architecture and designing a morphological processing module. This module is equipped to perform functions such as denoising, edge extraction, detail enhancement, and contrast amplification, as illustrated in Figure 2. Initially, the original image undergoes processes such as erosion, opening, top-hat, black-hat, and morphological gradient operations. Subsequently, images processed through the various operations are merged with the original image and fed into the backbone network, which can be described by Equation (8):

(8) $$G=concat(f,g_{2},g_{3},g_{5},g_{6},g_{7})$$

where f represents the original image and concat(⋅) denotes the operation of channel fusion. Given the tendency for image resolution to degrade with increased kernel dimensions, the kernel size is deliberately minimized to 3 × 3 to mitigate this effect. Furthermore, considering the dense distribution of naval vessels near coastal areas, ports, and riverways and the relatively small size of most ships within these images, dilation and closing operations are excluded from the morphological preprocessing module due to their predominantly adverse impacts.

### 2.2. Coordinate Channel Attention Module

**Figure 3:**
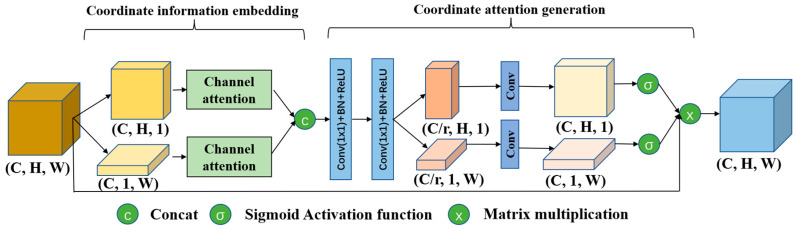
Coordinate Channel Attention Module.

In onshore scenarios, SAR imagery of ships is frequently marred by intricate backgrounds such as coastlines and ports, which significantly impede the accuracy of ship detection processes, leading to increasing rates of false positives and omissions. To address this challenge, this study proposes a Coordinate Channel Attention Module illustrated in Figure 3. This module synthesizes coordinate and channel attentions by embedding spatial coordinates into channels and subsequently generating coordinate-focused attention, thereby retaining essential positional information within the channel-based processing. The core concept of the Coordinate Channel Attention Module is to infuse spatial data into distinct channels and utilize channel-specific attentions to restore the positional details lost when channel attentions reduce a two-dimensional feature map to a singular representational point.

The embedding of coordinate information into channels is achieved by aggregating features along both the horizontal and vertical spatial directions of the feature map, followed by the application of channel attention. This approach not only captures long-range dependencies but also preserves precise positional information and enhances the weights of effective channels. For the input feature map $X=\left[x_{1},x_{2},\ldots ,x_{C}\right]\in R^{C\times H\times W}$, pooling kernels of sizes (*H*, 1) and (1, *W*) are employed to encode each channel along the horizontal and vertical coordinates, respectively. The output at the vertical position h for the c channel can be expressed as Equation (9):

(9) $$z_{c}(h)=\frac{1}{W}\sum_{i=1}^{W}x_{c}(h,i)$$

Similarly, the output of the vertical position w for the c channel can be expressed as Equation (10):

(10) $$z_{c}(w)=\frac{1}{H}\sum_{i=1}^{H}x_{c}(i,w)$$

Through the independent aggregation of features along the horizontal and vertical axes, the Coordinate Channel Attention Module adeptly incorporates positional data from both axes into distinct vector representations. Subsequently, these vectors are synthesized with channel attention mechanisms. This integration not only retains critical positional information but also facilitates the amplification of channel characteristics that are pivotal for the identification of ships, which substantially enhances the network’s capability to detect ships with higher accuracy.

In the coordinate attention generation module, spatial features derived from the horizontal and vertical axes are encoded via two distinct 1 × 1 convolution layers, generating attention maps for each respective dimension. These attention maps are subsequently employed to perform a weighted aggregation with the original feature map, thereby augmenting its sensitivity to the spatial coordinates of the target. Through the process of coordinate embedding, feature vectors along the horizontal $X^{\left(h\right)}\in R^{C\times H\times 1}$ and vertical $X^{\left(w\right)}\in R^{C\times 1\times W}$ dimensions are obtained. These vectors, by complementing each other dimensionally, effectively conserve the locational information of the target. Furthermore, in an effort to minimize computational demands, channel reduction is achieved using 1 × 1 convolution layers, along with the introduction of a hyperparameter r that denotes the rate of reduction, which is formulated as follows:

(11) $${\tilde{X}}^{\left(h\right)}=\mathrm{ReLU}\left(\mathrm{BN}\left({\mathrm{Conv}}_{1\times 1}\left(X^{\left(h\right)}\right)\right)\right)$$

(12) $${\tilde{X}}^{\left(w\right)}=\mathrm{ReLU}\left(\mathrm{BN}\left({\mathrm{Conv}}_{1\times 1}\left(X^{\left(w\right)}\right)\right)\right)$$

where Conv(·) is the 1 × 1 convolution layer, BN(·) is the batch normalization, and ReLU(·) is the activation function. The number of channels of the tensor sum is C/r, and r is set to 16 in the experiment. Then, the 1 × 1 convolution layer is used to restore the tensor ${\tilde{X}}^{\left(h\right)}$ and ${\tilde{X}}^{\left(w\right)}$ channel number to C in order to multiply the original feature map:

(13) $$g^{\left(h\right)}=\sigma \left(\mathrm{Conv}\left({\tilde{X}}^{\left(h\right)}\right)\right)$$

(14) $$g^{\left(w\right)}=\sigma \left(\mathrm{Conv}\left({\tilde{X}}^{\left(w\right)}\right)\right)$$

where $g^{\left(h\right)}$ and $g^{\left(w\right)}$ are the adaptive coordinate attention weights in the horizontal and vertical directions, respectively. Since $g^{\left(h\right)}$ and $g^{\left(w\right)}$ represent the weight and the value is between 0 and 1, the sigmoid activation function is used for the activation function. Finally, the output of the coordinate attention is Equation (15):

(15) $$y_{c}(i,j)=x_{c}(i,j)\times g_{c}^{\left(h\right)}(i)\times g_{c}^{\left(w\right)}(j)$$

In conclusion, the Coordinate Channel Attention architecture enhances the original coordinate attention mechanism by incorporating channel attention into the coordinate information embedding process. This enhancement effectively retains the spatial information while simultaneously emphasizing the salient features within the effective channels, thereby facilitating the network’s capability to accurately identify and localize targets. This refined attention mechanism is integrated into the feature extraction submodule of the CSPDarknet_CCA backbone network, as illustrated in Figure 1. Such integration is strategically designed to adeptly capture comprehensive ship-related information amidst complex backgrounds, thereby yielding more representative feature maps that significantly improve the network’s ship detection efficiency.

### 2.3. Four-Layer Bidirectional Feature Pyramid Network

This paper introduces a four-layer Bidirectional Feature Pyramid Network (FBFPN) depicted within the blue dashed box in Figure 1. Inspired by the structural philosophy of the Path Aggregation Network (PANet) [21], the FBFPN is engineered with a four-layer bidirectional information flow mechanism to achieve efficient feature fusion, which enables the network to recognize ships against complex backgrounds more effectively.

The FBFPN architecture proposed in this paper is constructed based on a dual-path approach, facilitating a bidirectional flow of information across different scales through the network hierarchy. The bottom-up path transmits high-resolution features with weaker semantic information to higher layers, while the top-down path enhances lower layers with semantically richer information. This bidirectional flow ensures the integration of rich contextual information, which is crucial for ship detection with various scales and complexity. In the top-down direction of the FBFPN, feature maps with 4✕, 8✕, 16✕, and 32✕ sampling are utilized. However, the 4✕ sampled feature maps, still carrying substantial background noise, are not used in designing the pyramid’s bottom-up path, which instead employs feature maps sampled at 8✕, 16✕, and 32✕.

Each level of the feature pyramid incorporates a fusion mechanism, where features from both the ascending and descending pathways are integrated through a meticulously designed combination of convolutional layers and nonlinear activation functions, which is expressed as follows:

(16) $${\tilde{X}}^{\left(w\right)}=SiLU\left(\mathrm{BN}\left({\mathrm{Conv}}_{3\times 3}(concat({\mathrm{Conv}}_{1\times 1}\left(X_{1}\right),X_{2})\right)\right)$$

where ${\mathrm{Conv}}_{1\times 1}(\cdot )$ is the 1 × 1 convolution layer, ${\mathrm{Conv}}_{3\times 3}(\cdot )$ is the 3 × 3 convolution layer, BN(·) is the batch normalization, *SiLU*(·) is the activation function, and concat represents channel merging. The design is based on the need to maintain the integrity of feature representations while facilitating the integration of diverse spatial and semantic cues. In the fusion process, we use a 1 × 1 convolution layer to adjust the channel dimension and then merge the corresponding features from both directions in the channel dimension. Afterward, a 3 × 3 convolution layer is applied to smooth the combined features and compress the feature dimensions, ensuring that the generated feature maps are robust to artifacts from the fusion of different feature maps.

Furthermore, we introduce lateral connections at each level of the pyramid, enhancing the direct flow of information and minimizing the loss of detail. These connections are optimized to convey detailed spatial information, which often becomes diluted in deeper network layers.

## 3. Experiment and Analysis

### 3.1. Dataset

In the experimental section, this study employs publicly available SAR ship detection datasets, namely the SAR Ship Detection Dataset (SSDD) [22] and the High-Resolution SAR Image Dataset (HRSID) [23], which contain ships across various scenes and multiple scales.

The SSDD is acquired using different polarization modes (HH, HV, VH, and VV) with sensors from RadarSat-2, TerraSAR-X, and Sentinel-1, featuring image resolutions ranging from 1 to 15 m. This dataset includes 1160 images, each approximately 500 × 500 pixels, and encompasses 2456 ships distributed across vast maritime and coastal regions. It offers a rich diversity in sea states, environmental conditions, and ship sizes and shapes, making it an invaluable resource for research. SSDD also contains challenging samples for SAR ship detection, such as small vessels with indistinct features, ships berthed in densely packed ports with overlapping hulls, and vessels under severe speckle noise. These complex scenarios are crucial for both traditional manual methods and modern deep-learning-based SAR ship detection research. Images in SSDD indexed with suffixes 1 and 9 are designated for the test set, while the remaining are used for training.

The HRSID, derived from Sentinel-1, TerraSAR-X, and TanDEM satellites, consists of 5604 images with 16,951 ships, averaging about three ships per image. The distribution within this dataset is diverse: small ships constitute 54.5%, mid-sized ships 43.5%, and large ships only 2%. These statistics not only reflect the distribution of ship sizes but also serve as a valuable reference for developing and refining ship detection algorithms. The HRSID targets high-traffic canals and trading ports, selected to ensure that high-resolution SAR images capture a variety of scenes within a limited field of view. For instance, a single image might show different types of ships in both offshore areas and cluttered anchorages, highlighting the challenges of detecting ships in scenarios often disturbed by man-made structures. This dataset emphasizes the difficulties in ship detection, particularly in offshore areas, and presents additional challenges for creating effective detection algorithms. Each image in HRSID averages 800 × 800 pixels and includes three polarization modes—HH, HV, and VV—covering a range of marine conditions and scenes from near-shore to offshore, thereby demonstrating significant diversity.

### 3.2. Evaluation Metric

To evaluate the effectiveness of the detection algorithm, this study employs the mean Average Precision (mAP), Precision (P), Recall (R), and F1 score as performance metrics. The computation formulas are as follows:

(17) $$\mathrm{Precision}=\frac{\mathrm{TP}}{\mathrm{TP}+\mathrm{FP}}$$

(18) $$\mathrm{Recall}=\frac{\mathrm{TP}}{\mathrm{TP}+\mathrm{FN}}$$

(19) $$\mathrm{mAP}=\int_{0}^{1}P(R)\mathrm{dR}$$

(20) $$F1=\frac{2\times \;\mathrm{Precision}\;\times \;\mathrm{Recall}\;}{\;\mathrm{Precision}+\mathrm{Recall}\;}$$

In this analysis, TP stands for the number of ships accurately detected, FP refers to the number of incorrect detections, and FN represents the ships that are not detected. The term P(R) signifies the Precision–Recall relationship curve. Precision measures the ratio of accurately predicted instances among all predictions made, whereas Recall quantifies the proportion of correctly identified and localized targets relative to the total number of actual targets. The F1 score evaluates the balance between Precision and Recall, being especially critical in scenarios where equal importance is placed on both. The mAP, which stands for mean Average Precision, assesses the average precision across a Recall spectrum from 0 to 1, while the F1 score is calculated as the harmonic mean of Precision and Recall. Higher mAP and F1 scores denote better detection performance. Additionally, APs, APm, and APl are metrics representing the detection accuracies for small, medium, and large targets, respectively; AP50 indicates the average precision at an Intersection over the Union (IOU) threshold of 0.5.

### 3.3. Experiment Settings

This study conducts experiments based on the Linux operating system and Pytorch 1.12.1 framework. The computational environment includes an Intel^®^ Xeon^®^ Gold 5118 CPU @ 2.3 GHz and an NVIDIA GeForce RTX 3090 GPU. All models mentioned in the ablation studies utilize the same SSDD. The training process spans 400 epochs, with a batch size of 8. An Adaptive Moment Estimation (Adam) optimizer is employed, featuring a cosine annealing schedule for learning rate reduction. The initial learning rate is set at 0.001, with a minimum rate of 0.00001. The score threshold of output is 0.3, and the IoU threshold of NMS is 0.5. For the SSDD and HRSID, the input image size is configured at 512 × 512 pixels and 800 × 800 pixels, respectively. The image sizes in the SSDD are inconsistent, and there is no fixed aspect ratio, which poses significant challenges for neural network training. Adjusting the aspect ratio of images to 1:1 can simplify the design of the network architecture and avoid the complexity of dealing with various sizes and proportions. Square images ensure that the network receives an equal amount of information in every direction while processing images, allowing for more uniform feature learning across the image. This helps improve the model’s learning efficiency and processing speed.

### 3.4. Ablation Study

#### 3.4.1. Experimental Analysis of Morphologic Processing Module Ablation

To analyze the impact of different morphological networks on ship images, ablation experiments were conducted based on the fundamental YOLOX network. These experiments included not only individual operations such as erosion, dilation, opening, closing, top-hat, black-hat, and morphological gradient but also combinations of different operations. Each experiment incorporated the original image, with results shown in Table 1.

**Figure 4:**
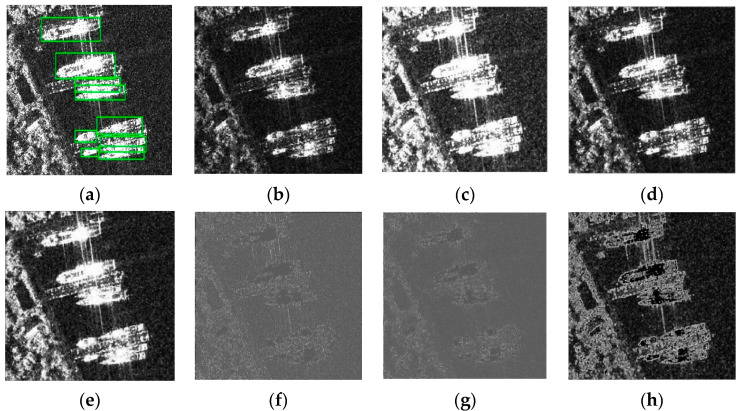
Different morphological effects: (**a**) Ground Truth; (**b**) Erosion; (**c**) Dilation; (**d**) Opening; (**e**) Closing; (**f**) Top-hat; (**g**) Black-hat; (**h**) Morphological Gradient. The green box indicates the ship position in the image.

Table 1 summarizes the impact of different morphological operations on detection performance, highlighting the significant improvements achieved with the opening operation. It is shown that among the separate morphological operations applied on the YOLOX-based network, only the closing operation decreased the detection mAP, whereas enhancements in the recall and F1 scores were observed with other operations, indicating their effectiveness in reducing omission errors in ship detection. Notably, the opening operation yielded the most significant improvement in mAP, increasing from 0.62% to 98.26%, attributable to its superior noise reduction and ability to segment dense ship clusters. Both top hat and morphological gradient operations achieved the highest increments in the recall, by 2.2%, enhancing ship features through detail and edge highlighting, respectively. Further experimentation with combinations of morphological operations indicated that not all combinations enhanced ship detection effectiveness. The impact of various morphological treatments on SAR ship images is illustrated in Figure 4.

Figure 4 illustrates the differential impact of morphological operations on ship detection. Erosion attenuates coastal background and speckle noise but concurrently reduces the saliency of ship features. Dilation not only amplifies ship characteristics but also intensifies background features and blurs the delineation between adjacent vessels. Opening operations effectively mitigate coastal and speckle noise while preserving essential ship features. Conversely, the closing operation does not significantly enhance ship imagery. Top hat processing accentuates ship detail features while simultaneously amplifying background interference. The black hat operation increases the contrast between ships and the background, enhancing detectability. Morphological gradient processing distinctly outlines ship edge features. Based on these observations, the designed morphological preprocessing module in this study excludes dilation and closing operations, only incorporating erosion, opening, top hat, black hat, morphological gradients, and the original image as the input into the primary network.

#### 3.4.2. The Ablation Experiments of Different Modules

Table 2 shows the ablation experiment results of different modules proposed in this paper on the baseline model YOLOX, which verifies the effectiveness of different modules for SAR ship detection. Relative to the YOLOX algorithm, the modules proposed in this study enhance the detection performance, increasing the mAP by 0.7%, 0.13%, and 0.41%, respectively, with a cumulative increase of 0.83% when all three modules are employed. The morphological module reduces the impact of speckle noise and accentuates the edges and detail features of ships, effectively delineating boundaries in dense targets and distinguishing these targets from the complex surrounding environment, thereby aiding in the network’s detection capabilities. The CCA attention module integrates channel, spatial, and directional information, thus enhancing the salient features of ships within the feature maps, which significantly enhances the accuracy of the network’s target recognition. This results in notable improvements in accuracy and F1 scores, with increases of 2.03% and 1.65%, respectively. The FBFPN enhances the coverage of the existing three-layer feature pyramid by incorporating larger scale feature maps into the pyramid network, capturing intricate ship details, and enhancing detection performance in complex scenes. Among all the modules, FBFPN shows the most substantial increase in the F1 score, with an improvement of 1.86%.

**Figure 5:**
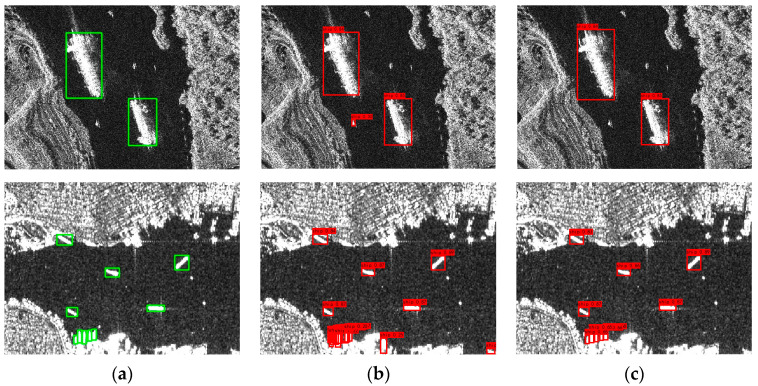
Partial image detection results of SSDD: (**a**) Ground Truth; (**b**) YOLOX; (**c**) ours. The green box indicates the actual position of the ship, and the red box indicates the detection result.

To verify that the improvements to the algorithm indeed enhance the ship detection performance, this paper selected a subset of images from the SSDD, as shown in Figure 5, which included near-shore targets, offshore targets, and targets of varying sizes. Figure 5a presents images with true labels, while Figure 5b,c display the target detection results from the YOLOX algorithm and the algorithm developed in this study, respectively. As observed in Figure 5b, the original algorithm is susceptible to speckle noise when detecting small targets, leading to false alarms; furthermore, due to insufficient feature extraction of the original algorithm, the detection of near-shore targets is poor, with weak boundary resolution capabilities, thus prone to missed detections and false alarms. This study addressed these issues by incorporating a morphological processing module, a coordinate channel attention module, and a Feature Balanced Feature Pyramid Network. As shown in Figure 5c, the algorithm developed in this study achieves higher accuracy in ship detection.

### 3.5. Comparison with the State-of-the-Art Methods

#### 3.5.1. Compared with the Classical Target Detection Algorithm

To quantitatively assess the detection performance of the method proposed in this paper, a comparison was first made with classic object detection methods. These methods include Cascade R-CNN [24], Libra R-CNN [25], FCOS [26], CenterNet [27], YOLOX [28], and YOLO v7 [29]. Table 3 presents the results of these methods on the SSDD and HRSID. The algorithm discussed in this paper exhibited superior performance on both datasets, particularly on the SSDD, where the mAP increased by 4.4%, 5.64%, 4.76%, 4.92%, 0.7%, and 0.48% compared to other algorithms, while also showing good results in terms of F1 score, recall, and accuracy. The accuracy improvement likely results from the morphological processing that enhances ship boundary and detail features and also provides a denoising effect. The proposed algorithm achieves the highest mAP, indicating the best detection performance among the compared algorithms. Although CenterNet demonstrates a significant advantage in recall on the HRSID, its mAP is low.

**Figure 6:**
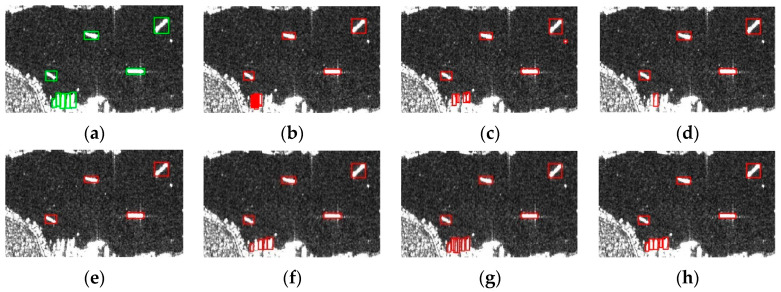
Detection results of the proposed method and classical target detection algorithm (SSDD): (**a**) Ground Truth; (**b**) Cascade R-CNN; (**c**) Libra R-CNN; (**d**) FCOS; (**e**) CenterNet; (**f**) YOLOX; (**g**) YOLOv7; (**h**) Ours. The green box indicates the actual position of the ship, and the red box indicates the detection result.

**Figure 7:**
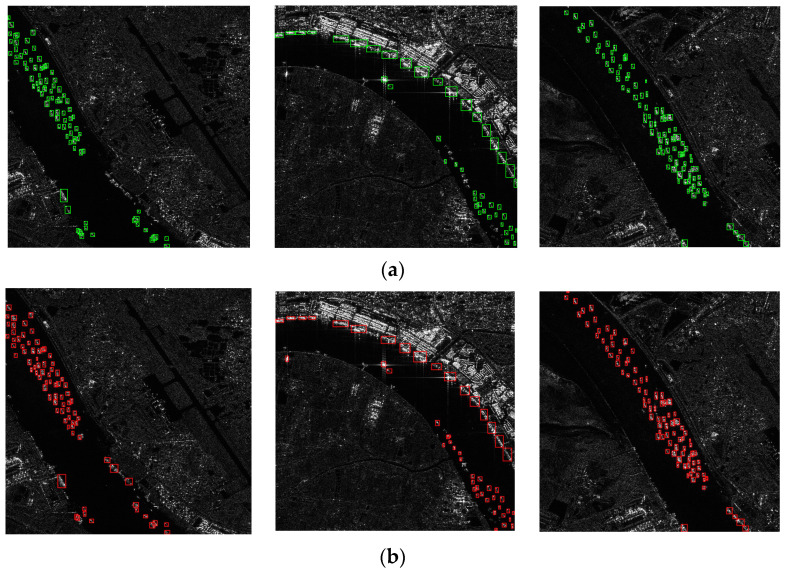
Our method detects the results on the HRSID. (**a**) Ground Truth; (**b**) ours. The green box indicates the actual position of the ship, and the red box indicates the detection result.

Figure 6 shows the detection results of selected images from the SSDD using different algorithms, highlighting that the proposed method accurately detects near-shore ships in complex backgrounds, whereas Cascade R-CNN and Yolov7 have more false alarms and other methods make more missed detections. Figure 7 shows the detection results of selected images from the HRSID using the proposed method, demonstrating effective target detection in scenarios with complex scenes, small targets, and speckle noise, thus validating the effectiveness of the method.

#### 3.5.2. Compared with Other Improved SAR Ship Detection Algorithms

To further validate the advanced nature of the proposed algorithm, it was compared with other deep learning-based SAR ship detection methods, including FoveaBox [30], FBR-Net [31], LPEDet [32], HRSDNet [23], and FBUA-Net [33], the results are shown in Table 4. The results showed that the method described in this paper outperforms most of the other methods. Specifically, in the SSDD, the AP50 metrics improved by 6.3%, 3.4%, 3.6%, and 1.3%, respectively. As the network leverages the YOLOX framework, it inherently benefits from a lightweight effect; thus, the model size is significantly smaller than other networks. However, the morphological operation module processes the image in multiple ways, which consumes a lot of time, resulting in lower frames per second (FPS) for the model. The performance of the proposed method in the HRSID is not optimal, possibly because the HRSID contains many small ships, which are easily affected by certain morphological operations.

## 4. Conclusions

This paper introduces a ship detection method that combines morphological operations with convolutional neural networks, effectively addressing prevalent issues such as speckle noise and complex backgrounds in remote sensing images. Unlike prior studies, the primary advantage of this approach is the integration of morphological preprocessing with advanced deep learning techniques. The morphological preprocessing module developed here not only significantly reduces background noise but also enhances the depiction of ship edges and detailed features, aspects seldom tackled in earlier research. Additionally, the newly introduced coordinate channel attention module markedly improves the network’s sensitivity across spatial dimensions and channels, thereby boosting the accuracy of ship localization and identification. The four-layer bidirectional feature pyramid network further enhances the model’s capability to capture detailed features, substantially improving performance in complex scenarios.

However, this method also exhibits certain limitations. Firstly, the addition of multiple modules increases the overall parameter count of the model, potentially affecting operational efficiency and practical deployment. Secondly, despite this method’s excellent performance in ship detection, the profound enhancement of algorithmic mechanisms and exploration of theoretical foundations remain inadequate. Future research should focus on further investigating these areas.

## Figures and Tables

**Table 1:** Morphologic ablation experiments on SSDDs (best results shown in bold). The checkmark represents the morphological network layer used.

Erosion	Dilation	Opening	Closing	Top-Hat	Black-Hat	Morphological Gradient	Precision (%)	Recall (%)	F1 (%)	mAP (%)
							95.34	93.77	94.55	97.64
✓							95.94	95.24	95.59	98.24
	✓						95.05	95.05	95.05	97.67
		✓					**96.85**	95.60	**96.22**	98.27
			✓				95.18	93.96	94.56	97.25
				✓			95.80	**95.97**	95.88	98.13
					✓		95.26	95.60	95.43	98.24
						✓	96.32	**95.97**	96.15	98.26
✓	✓						96.27	94.51	95.38	98.07
		✓	✓				95.22	94.87	95.05	97.45
				✓	✓		96.11	95.05	95.58	98.24
✓	✓	✓	✓	✓	✓	✓	95.10	**95.97**	95.53	98.05
✓		✓		✓	✓	✓	96.46	94.69	95.56	**98.34**

**Table 2:** Ablation experiments of different modules on the SSDD (best results shown in bold). The checkmark indicates the network module used.

Morphological	CCA	FBFPN	mAP (%)	F1 (%)	Recall (%)	Precision (%)	AP_s_ (%)	AP_m_ (%)	AP_l_ (%)
			97.64	94.55	93.77	95.34	54.4	67.0	63.5
✓			98.34	95.56	94.69	96.46	**55.6**	66.7	67.7
	✓		97.77	96.20	95.05	**97.37**	54.2	67.9	**67.9**
		✓	98.05	**96.41**	95.79	97.03	55.1	67.5	65.0
✓	✓	✓	**98.47**	95.88	**95.97**	95.80	54.0	**68.0**	64.4

**Table 3:** Objective evaluation of seven classic detection methods (best results shown in bold).

Method	Dataset	Precision (%)	Recall (%)	F1 (%)	mAP (%)	Parameters (M)	FLOPs (G)
Cascade RCNN [24]	SSDD	94.07	93.04	94	94.07	87.9	110.5
Libra R-CNN [25]	SSDD	90.49	90.60	91	92.83	60.4	83.0
FCOS [26]	SSDD	94.98	90.11	92	93.71	50.8	69.8
CenterNet [27]	SSDD	94.82	93.96	94	93.55	20.2	25.9
YOLOX-s [28]	SSDD	96.57	92.86	95	97.77	**8.94**	**17.1**
YOLOv7 [29]	SSDD	**96.91**	91.94	94	97.99	37.2	33.6
ours	SSDD	95.80	**95.97**	**96**	**98.47**	9.86	38.21
Cascade RCNN [24]	HRSID	86.96	85.53	86	84.79	87.9	209.7
Libra R-CNN [25]	HRSID	81.14	86.24	84	86.67	60.4	182.6
FCOS [26]	HRSID	88.66	82.05	85	86.68	50.8	170.6
CenterNet [27]	HRSID	87.22	**87.53**	87	88.09	20.2	63.3
YOLOX-s [28]	HRSID	92.41	84.62	**89**	91.25	**8.94**	**41.81**
YOLOv7 [29]	HRSID	90.10	84.47	87	91.29	37.2	82.1
ours	HRSID	**92.67**	84.84	**89**	**91.71**	9.86	93.3

**Table 4:** Objective evaluation of different SAR ship detection methods (best results shown in bold).

Method	Dataset	AP50 (%)	Parameters (M)	FLOPs (G)	FPS (img/s)
FoveaBox [30]	SSDD	91.2	36.01	73.12	**42.9**
FBR-Net [31]	SSDD	94.1	32.5	-	24.9
HRSDNet [23]	SSDD	93.9	-	-	9.9
FBUA-Net [33]	SSDD	96.2	36.54	71.11	31.8
ours	SSDD	**97.5**	9.86	38.21	14.3
FoveaBox [30]	HRSID	85.9	36.01	199.43	20.5
LPEDet [32]	HRSID	89.7	**5.68**	**18.38**	**24.9**
HRSDNet [23]	HRSID	88.4	-	-	7.9
FBUA-Net [33]	HRSID	**90.3**	36.54	194.12	12.2
ours	HRSID	90.0	9.86	93.27	11.9

## Data Availability

Data are contained within the article.
